# Microbial Necromass and Extracellular Enzyme Activities Are Associated with Depth-Dependent Soil Carbon Stabilization Along a Wildfire-Severity Gradient

**DOI:** 10.3390/microorganisms14061380

**Published:** 2026-06-22

**Authors:** Shaqian Liu, Rui Yang

**Affiliations:** 1 Key Laboratory of Plant Resource Conservation and Germplasm Innovation in Mountainous Region (Ministry of Education), College of Life Sciences, Institute of Agro-Bioengineering, Guizhou University, Guiyang 550025, China; liu5771998@163.com; 2 College of Forestry, Guizhou University, Guiyang 550025, China

**Keywords:** wildfire gradient, microbial necromass, soil carbon stabilization, extracellular enzymes, CAZy functions, depth dependence

## Abstract

Wildfire can alter soil organic carbon (SOC) pools and microbial pathways of carbon stabilization, but depth-dependent links between microbial necromass and stable carbon pools remain unclear. We investigated a wildfire-severity gradient in a subtropical coniferous forest in Guizhou, China, including four severity classes (unburned, light, moderate, and severe) and two soil layers (0–20 and 20–40 cm). We measured easily oxidizable organic carbon (EOC), recalcitrant organic carbon (ROC), SOC, amino sugars, microbial necromass carbon (MNC), extracellular enzyme activities, and carbohydrate-active enzyme (CAZy) functional indices. MNC peaked under moderate wildfire in both layers, increasing by 73.8% and 85.1% in the topsoil and subsoil, respectively, relative to unburned plots. After accounting for soil physicochemical properties and wildfire severity, MNC was more strongly associated with ROC and SOC in the topsoil than in the subsoil. Extracellular enzyme activities were positively associated with amino sugars and necromass pools, whereas CAZy composite indices showed weaker relationships that did not persist after false discovery rate correction. Exploratory path analysis suggested that the EOC–NAG–MNC–ROC–SOC chain was more pronounced in the topsoil, while the subsoil showed weaker chained associations and stronger direct EOC–MNC and EOC–ROC links. Overall, microbial necromass was associated with depth-dependent post-fire carbon stabilization.

## 1. Introduction

Wildfire is a major disturbance shaping forest structure, ecosystem functioning, and terrestrial carbon cycling. Under global warming and increasing human disturbance, the frequency, extent, and severity of wildfires have increased in many regions, raising concern that forest soils may shift from carbon sinks to carbon sources [1,2]. Fire can cause immediate carbon losses through combustion of aboveground vegetation and surface organic layers, leading to short-term declines in soil organic carbon (SOC) and microbial biomass carbon (MBC). At the same time, pyrogenic carbon (PyC), together with fire-induced changes in soil aggregates and mineral interfaces, may contribute to the persistence of certain organic matter fractions [2]. Previous studies have shown that wildfire effects on SOC and its fractions vary with wildfire severity, post-fire recovery stage, and ecosystem type [1,3]. However, the microbial pathways associated with post-fire soil carbon stabilization, particularly their variation across soil depths, remain insufficiently understood.

Cross-ecosystem studies have shown that microbial necromass carbon (MNC) contributes substantially to SOC and represents an important component of long-term soil carbon sequestration [4,5,6,7]. Within this framework, plant residues are better viewed as precursor carbon sources because they are first assimilated, decomposed, and resynthesized by microorganisms before being transformed into microbial necromass and subsequently stabilized through interactions with minerals or soil aggregates. Amino sugars such as glucosamine (GluN) and muramic acid (MurA), which are mainly derived from fungal chitin and bacterial peptidoglycan, are therefore widely used as molecular indicators of microbial necromass contributions to SOC [8,9]. Under wildfire conditions, however, it remains unclear how wildfire severity and soil depth are associated with the coupling between MNC and different carbon pools, including easily oxidizable organic carbon (EOC), recalcitrant organic carbon (ROC), and SOC.

From the perspective of carbon fractions, EOC is generally regarded as a fast-turnover and disturbance-sensitive active carbon pool, whereas ROC represents a slower-turnover and relatively stable component of SOC. A commonly proposed framework suggests that EOC is first assimilated by microorganisms and transformed into microbial biomass through extracellular enzymatic processes; following microbial death, cell-wall residues contribute to MNC, which may subsequently become associated with relatively stable carbon fractions such as ROC and SOC through mineral interactions, aggregate protection, or pyrogenic carbon matrices [5,8,9]. Fire may reshape this framework by altering EOC supply, enzyme activity patterns, necromass accumulation, and PyC formation. However, whether this EOC–enzyme activity–MNC–ROC/SOC framework shows clear depth dependence under post-fire conditions remains poorly quantified.

Extracellular enzymes and carbohydrate-active enzyme genes (CAZymes) provide two complementary perspectives on carbon transformation. Extracellular enzyme activities reflect the realized intensity of organic matter decomposition and nutrient cycling, whereas CAZy families indicate the potential capacity of microbial communities to degrade or synthesize substrates such as cellulose, hemicellulose, chitin, and extracellular polysaccharides [10,11]. Previous metagenomic studies have shown that CAZy composition varies among vegetation types and along degradation or restoration gradients, driven jointly by vegetation characteristics and SOC fractions [10,11]. However, functional gene abundance represents a potential functional reservoir rather than realized process intensity, and it does not necessarily correspond linearly with measured enzyme activities or necromass accumulation. Accordingly, evidence linking CAZy functional potential, extracellular enzyme activity, amino sugars, MNC, and stable carbon pools across wildfire gradients and soil profiles remains limited.

Against this background, we investigated a subtropical coniferous forest in Guizhou, China, along a wildfire gradient spanning four wildfire-severity classes (unburned, light, moderate, and severe) and two soil layers (topsoil layer, 0–20 cm; subsoil layer, 20–40 cm). We quantified EOC, ROC, and SOC fractions, amino sugars and MNC, extracellular enzyme activities, and CAZy functional indices. Specifically, we asked two questions: (1) How are wildfire severity and soil depth associated with the coupling between MNC and different carbon pools (EOC, ROC, and SOC)? (2) Does the EOC–NAG–MNC–ROC/SOC framework show depth-dependent association patterns, and how do extracellular enzyme activities and CAZy functions relate to this framework? By addressing these questions, this study aims to provide an observational basis for understanding depth-dependent associations in post-fire soil carbon stabilization in subtropical coniferous forests.

## 2. Materials and Methods

### 2.1. Study Area

**Figure 1:**
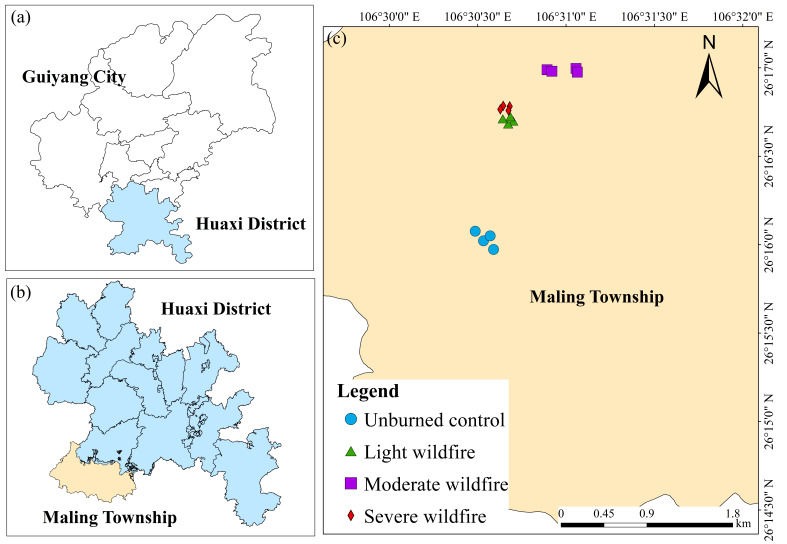
Location of the study area and sampling sites along the wildfire-severity gradient in Maling Township, Guizhou Province, China. (**a**) Location of Huaxi District within Guiyang City, Guizhou Province, China. (**b**) Location of Maling Township within Huaxi District. (**c**) Spatial distribution of sampling sites representing the four wildfire-severity classes in Maling Township, including unburned control, light wildfire, moderate wildfire, and severe wildfire. The map was generated using ArcGIS 10.7 (Esri, Redlands, CA, USA).

The study was conducted in Maling Township, Huaxi District, Guiyang City, Guizhou Province, China (26°13′28.87″–26°19′16.69″ N, 106°28′49.47″–106°37′51.32″ E) (Figure 1). The region has a subtropical plateau monsoon climate, with a mean annual temperature of 14.5 °C (January mean 4.5 °C; July mean 23.5 °C), a frost-free period of approximately 210 days, and a mean annual precipitation of 1150 mm. Elevation ranges from 999 to 1573 m. The soils are locally classified as yellow soils in China and correspond to Acrisols in the World Reference Base for Soil Resources (WRB) system [12].

### 2.2. Wildfire Severity Classification

**Figure 2:**
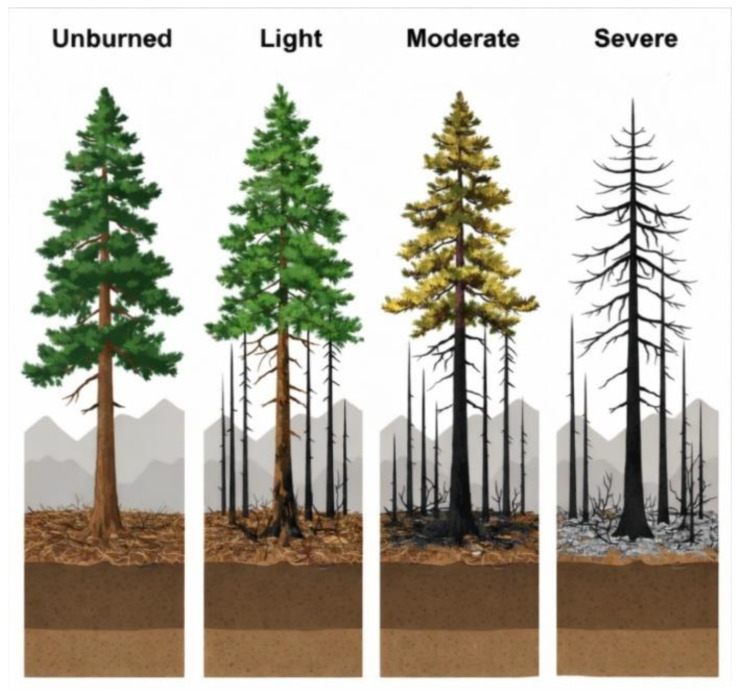
Conceptual illustration of wildfire severity classes used in this study.

Plots were categorized into four observed wildfire severity classes along the wildfire gradient, namely unburned (CK), light (L), moderate (M), and severe (S), based on integrated field indicators of fire behavior and burn effects on vegetation and soil (Table 1; Figure 2). The classification considered: (i) fire type (surface fire vs. crown fire), (ii) proportion of burned woody material (≤30%, 30–70%, and ≥70%), (iii) flame height (<1.5, 1.5–3.0, and >3.0 m), (iv) canopy and trunk damage, and (v) soil organic-layer condition, including the extent of charring and ash deposition. Light wildfire plots were characterized by surface fire, limited woody combustion, low flame height, scorched trunks with largely green canopies, and an intact organic layer with only millimeter-scale charring. Moderate wildfire plots showed intermediate burned-wood proportion and flame height, with scorched trunks and largely green canopies. Severe wildfire plots showed combined surface and crown fire, extensive woody combustion, high flame height, burned crowns without remaining green leaves, and centimeter-thick ash or charred organic deposits.

### 2.3. Fire Event, Plot Background, and Study Design

Information on wildfire occurrence, sampling time, and stand background was obtained from local forest management records and verified through field surveys (Table 1). The burned area was affected by a wildfire event in February 2024, and the field investigation was conducted in June 2024, approximately four months after the fire. Within the wildfire-affected landscape, four wildfire-severity classes were selected, namely unburned control (CK), light wildfire (L), moderate wildfire (M), and severe wildfire (S). For each wildfire-severity class, four spatially separated sampling points were established, with the distance between adjacent sampling points exceeding 40 m to capture local spatial heterogeneity. All study stands were planted plantations located within the same regional climatic zone. To reduce broad-scale site heterogeneity, the sampling points were selected within the same geographic region and under generally comparable climatic conditions, and their key topographic attributes, including representative elevation, slope aspect, and slope, are summarized in Table 1. Because the study was conducted within a single wildfire-affected landscape rather than across multiple independent wildfire events, the results should be interpreted as an observational comparison along a wildfire-severity gradient rather than as evidence from a fully replicated field experiment.

### 2.4. Soil Sampling

Soil sampling was conducted in June 2024, approximately four months after the wildfire event. Wildfire severity class definitions followed the criteria described above and summarized in Table 1. For each wildfire-severity class, four spatially separated sampling points were established, with the distance between adjacent sampling points exceeding 40 m to capture local spatial heterogeneity. At each sampling point, soil samples were collected from two depth intervals: 0–20 cm and 20–40 cm. For each sampling point and soil depth, 3–5 soil cores were collected from the surrounding area and thoroughly homogenized to obtain one composite sample. Surface litter was carefully removed before coring to minimize exogenous contamination. Soil cores were collected sequentially from deeper to shallower layers, and visible stones, coarse fragments, and plant residues such as roots were removed during sample processing. In total, 32 composite soil samples were obtained, including 4 wildfire-severity classes × 4 sampling points × 2 soil depths. Fresh soil samples were immediately labeled in the field, placed into sealed bags or sterile tubes, transported to the laboratory under cooled conditions, and then subdivided according to the analytical requirements for physicochemical properties, carbon-fraction measurements, enzyme assays, and necromass analyses. Subsamples for metagenomic analysis were placed in sterile tubes and stored at −80 °C until DNA extraction and subsequent shotgun metagenomic sequencing.

### 2.5. Soil Physicochemical Properties and Carbon Fractions

Soil physicochemical properties included soil moisture content (SMC), bulk density (BD), pH, soil total nitrogen (STN), soil total phosphorus (STP), soil total potassium (STK), soil alkali-hydrolyzable nitrogen (SAN), soil available phosphorus (SAP), soil available potassium (SAK), nitrate nitrogen (NO_3_^−^-N), and ammonium nitrogen (NH_4_^+^-N). SMC was determined gravimetrically by oven-drying fresh soil to constant mass at 105 °C, BD by the undisturbed core (ring-knife) method, and pH using a pH meter in a soil–water suspension [15]. STN was determined by the diffusion method (ammonia diffusion after oxidation), STP by acid extraction followed by molybdenum–antimony colorimetry, and STK by acid extraction followed by flame photometry. SAN was determined by alkaline hydrolysis followed by diffusion, SAP by HCl–H_2_SO_4_ extraction followed by molybdenum–antimony colorimetry, and SAK by 1 mol L^−1^ ammonium acetate extraction followed by flame photometry [15]. NO_3_^−^-N and NH_4_^+^-N were determined using commercial assay kits (Solarbio, Beijing, China) according to the manufacturer’s instructions.

The measured carbon fractions included soil organic carbon (SOC), dissolved organic carbon (DOC), microbial biomass carbon (MBC), easily oxidizable organic carbon (EOC), and recalcitrant organic carbon (ROC). SOC was determined by potassium dichromate oxidation with external heating following the modified Walkley–Black method [16]. DOC was extracted from fresh soil with distilled water at a soil ratio of 1:5 at 25 °C for 1 h, using a water-extraction procedure modified from a previously described method [17]. This procedure represented room-temperature water extraction rather than hot-water extraction. The extracts were then centrifuged, filtered through a 0.45 μm membrane, and measured using a TOC analyzer. MBC was measured using the chloroform fumigation–extraction method [18]. EOC was determined using the KMnO_4_ oxidation method [19], and ROC was operationally defined for each sample as SOC minus EOC prior to downstream statistical analyses [19]. DOC and MBC analyses were conducted by Guizhou Wela Detection Technology Co., Ltd. (Guizhou, China). Descriptive statistics for soil physicochemical properties and carbon fractions are provided in Tables S1 and S2, respectively.

### 2.6. Amino Sugars and Microbial Necromass Carbon

Soil amino sugars, including glucosamine (GluN), galactosamine (GalN), and muramic acid (MurA), were determined using a pre-column derivatization high-performance liquid chromatography (HPLC) method. Briefly, dried soil samples were hydrolyzed with HCl at 105 °C for 6 h, purified, derivatized with 1-phenyl-3-methyl-5-pyrazolone (PMP) reagent, and then analyzed using an Agilent 1260II HPLC system equipped with a diode-array detector (DAD) at 250 nm. The mobile phase consisted of acetonitrile and 0.1 mol L^−1^ ammonium acetate, with compound-specific ratios of 22:78 for GalN, 25:75 for MurA, and 20:80 for GluN. The injection volume was 5 μL. External standard solutions were used for calibration, and amino sugar concentrations were quantified based on peak areas. The analytical precision requirement was a relative difference of less than 15%.

Microbial necromass carbon was estimated from amino sugar contents according to Appuhn and Joergensen [20], as widely adopted in subsequent studies. Bacterial necromass carbon (BNC) was estimated from MurA, fungal necromass carbon (FNC) was estimated from GluN after correction for the bacterial contribution, and total microbial necromass carbon (MNC) was calculated as the sum of FNC and BNC:

(1) $$FNC=\left(C_{GluN}/179.17-2\times C_{MurA}/251.23\right)\times 179.17\times 9$$

(2) $$BNC=C_{MurA}\times 45$$

(3) MNC=FNC+BNC

where MNC is the total microbial necromass C content (mg C·kg^−1^); FNC is the fungal necromass C content (mg C·kg^−1^); BNC is the bacterial necromass C content (mg C·kg^−1^); C_GluN_ is the glucosamine content (mg·kg^−1^); C_MurA_ is the muramic acid content (mg·kg^−1^); and 179.17 and 251.23 are the molecular weights of GluN and MurA, respectively.

### 2.7. Extracellular Enzyme Assays

The activities of β-glucosidase (BG), N-acetylglucosaminidase (NAG), cellobiohydrolase (CBH), cellulase, and polyphenol oxidase (POX) were measured using commercial biochemical assay kits obtained from Beijing Solarbio Science & Technology Co., Ltd. (Beijing, China). Enzyme assays were conducted for all 32 soil samples, corresponding to four wildfire-severity classes, four sampling points within each wildfire-severity class, and two soil layers. All assays were performed using air-dried soil samples according to the manufacturer’s protocols. Soil extracts and reaction systems were prepared from air-dried soil, and enzyme activities were calculated using the equations provided with each kit. Activity values were expressed on an air-dried soil mass basis for subsequent statistical analyses.

### 2.8. Metagenomic Sequencing and CAZy Annotation

#### 2.8.1. DNA Extraction, Library Preparation, and Metagenomic Sequencing

Total genomic DNA was extracted from soil samples using the Mag-Bind^®^ Soil DNA Kit (Omega Bio-tek, Norcross, GA, USA) according to the manufacturer’s instructions. DNA concentration and purity were measured using a TBS-380 fluorometer (Turner BioSystems, Sunnyvale, CA, USA) and a NanoDrop 2000 spectrophotometer (Thermo Fisher Scientific, Waltham, MA, USA), respectively. DNA integrity was assessed by 1% agarose gel electrophoresis. In total, 32 soil samples were subjected to shotgun metagenomic sequencing, including four wildfire-severity classes (unburned, light, moderate, and severe), four sampling points within each wildfire-severity class, and two soil layers (0–20 and 20–40 cm).

Qualified DNA was fragmented to an average size of approximately 350 bp using a Covaris M220 ultrasonicator (Covaris, Woburn, MA, USA) for paired-end library preparation. Subsequently, paired-end libraries were constructed using NEXTFLEX Rapid DNA-Seq (Bioo Scientific, Austin, TX, USA). Because shotgun metagenomic sequencing was used, no locus-specific PCR primers or bacteria-/fungi-specific marker regions were targeted during library preparation. Libraries were sequenced on an Illumina NovaSeq platform (Illumina Inc., San Diego, CA, USA) at Majorbio Bio-Pharm Technology Co., Ltd. (Shanghai, China) to generate 2 × 150 bp paired-end reads using the NovaSeq 6000 S4 Reagent Kit v1.5 (300 cycles; Illumina Inc., San Diego, CA, USA). The raw metagenomic sequence data have been deposited in the NCBI Sequence Read Archive (SRA) under BioProject accession number PRJNA1280738 and SRA study accession number SRP593774.

#### 2.8.2. Quality Control, Assembly, and Gene Catalog Construction

Raw sequence data were processed using the Majorbio Cloud Platform (www.majorbio.com) (accessed on 18 April 2026). Briefly, paired-end Illumina reads were processed to remove adapter sequences, and low-quality reads (length < 50 bp or with a quality score < 20) were removed using fastp v0.23.0 [21]. After quality control, 6.14–8.31 Gb of clean bases were obtained per sample (Table S3). High-quality metagenomic reads were assembled using MEGAHIT v1.1.2 [22]. High-quality reads from each sample were assembled separately (i.e., no co-assembly across samples was performed), and assembly statistics, including contig number, total contig length, and N50, were calculated for each assembly (Table S3). The resulting contigs from all samples were subsequently pooled for downstream gene prediction and construction of a non-redundant gene catalog. Contigs with a length ≥ 300 bp were retained in the final assembly and subsequently used for gene prediction and functional annotation.

#### 2.8.3. ORF Prediction, Non-Redundant Gene Catalog Construction, and Abundance Estimation

Open reading frames (ORFs) were predicted from assembled contigs using Prodigal v2.6.3 [23]. Predicted ORFs with a length ≥ 100 bp were retrieved and translated into amino acid sequences using EMBOSS v6.6.0 [24] with the NCBI translation table.

To construct a non-redundant gene catalog, all predicted ORFs from all samples were clustered using CD-HIT v4.6.1 [25] with thresholds of 90% sequence identity and 90% coverage. Coverage was calculated based on the shorter sequence in each pairwise comparison, and the longest ORF in each cluster was retained as the representative sequence. High-quality reads from each sample were then aligned to the non-redundant gene catalog using SOAP v2.21 [26] with an alignment identity threshold of 95% to estimate gene abundance. The resulting gene-abundance matrix was used for downstream functional annotation and calculation of the composite functional indices.

#### 2.8.4. CAZy Annotation and Composite Functional Indices

Carbohydrate-active enzyme (CAZy) annotation was performed on the representative sequences in the non-redundant gene catalog using hmmscan against the CAZy database with an e-value cutoff of 1 × 10^−5^. Based on the annotated CAZy family abundances, four composite functional indices were constructed to represent chitin/chitosan degradation, peptidoglycan degradation, extracellular polysaccharide synthesis, and overall carbohydrate depolymerization potential. The assignment of individual CAZy families to each index is presented in Table S8. Before index construction, the abundance of each family was standardized across samples, and the standardized values were averaged within each functional category. The resulting sample-level values of the four CAZy composite functional indices are provided in Table S9.

### 2.9. Statistical Analysis

Because each wildfire severity class was represented within a single wildfire-affected landscape, all statistical analyses were intended to describe patterns among within-severity sampling points along the observed wildfire-severity gradient. The results should therefore be interpreted as observational patterns rather than as independently replicated treatment effects of wildfire severity.

For soil physicochemical properties, carbon fractions, amino sugars, microbial necromass carbon, and extracellular enzyme activities, models included wildfire severity (Wildfire), soil depth (Depth), and their interaction (Wildfire × Depth) as fixed effects. Sampling-point identity (SamplingPointID) was initially included as a random intercept to account for paired sampling of the two soil layers within each spatially separated sampling point. When models showed convergence problems or singular fits, they were simplified to fixed-effects linear models. Variables with skewed distributions were log_10_(x + 1) transformed when necessary.

Estimated marginal means (EMMs) were used for post hoc comparisons among wildfire-severity classes within each soil layer and between soil layers within each wildfire-severity class. *p* values from multiple comparisons were adjusted using the Benjamini–Hochberg false-discovery-rate (BH-FDR) procedure. Effect sizes were reported as partial η^2^ where appropriate.

To describe the shape of MNC variation along the ordered wildfire-severity gradient, generalized additive models (GAMs) were fitted separately for the two soil layers, with severity coded from 0 to 3. These analyses were considered exploratory descriptions of nonlinear patterns and were not used as evidence of causal threshold responses.

Depth-stratified associations between MNC and stable carbon pools were evaluated using standardized multiple regression. Within each soil layer, ROC or SOC was used as the response variable, and standardized MNC was used as the focal predictor. Soil pH, BD, SMC, DOC, MBC, and wildfire severity were included as covariates where applicable. Nested model comparisons and partial R^2^ were used to evaluate the additional explanatory contribution of MNC beyond the covariates.

Associations among CAZy composite indices, extracellular enzyme activities, amino sugars, and necromass pools were examined using depth-stratified partial correlations while controlling for pH, BD, and SMC. *p* values were adjusted within each soil layer using the BH-FDR procedure. Partial-correlation networks were used only to visualize robust covariation structures and were interpreted as exploratory rather than causal.

Finally, the EOC-NAG-MNC-ROC/SOC framework was evaluated using depth-stratified path analysis based on standardized piecewise linear equations. The standardized path coefficients and chained indirect effects were used for relative comparison between soil layers. Given the limited sample size and non-replicated wildfire-severity design, these path analyses were intended to illustrate potential depth-dependent association structures rather than establish definitive causal pathways.

Unless otherwise stated, all analyses were performed in R 4.4.3 using the packages tidyverse, car, emmeans, mgcv, ppcor, and piecewiseSEM. Statistical significance was assessed at *p* < 0.05.

## 3. Results

### 3.1. Responses of MNC Pools to Wildfire Severity and Soil Depth

**Figure 3:**
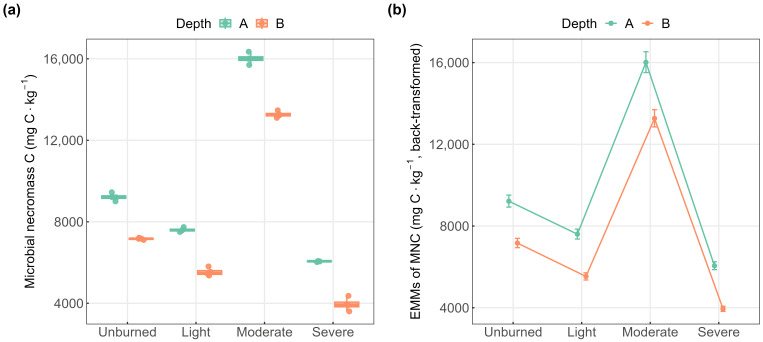
Effects of wildfire severity and soil depth on MNC. (**a**) Observed MNC values; (**b**) estimated marginal means with 95% confidence intervals. A and B indicate the 0–20 cm and 20–40 cm soil layers, respectively.

MNC varied markedly across wildfire severity, soil layers, and their interaction (Figure 3; Table S4). The corresponding test statistics were large for wildfire severity (F = 1629.146, partial η^2^ = 0.995), soil depth (F = 665.596, partial η^2^ = 0.965), and the wildfire severity × depth interaction (F = 19.889, partial η^2^ = 0.713), indicating that variation in MNC among wildfire severity classes exceeded that between soil layers and that this variation differed between depths.

Across both soil layers, MNC was highest in the moderate-wildfire plots and lowest in the severe-wildfire plots (Figure 3; Table S5). In the topsoil layer, MNC reached 16,012.89 mg C kg^−1^ under moderate wildfire, representing a 73.8% increase relative to the unburned plots. In the subsoil layer, MNC reached 13,266.63 mg C kg^−1^ under moderate wildfire, representing an 85.1% increase relative to the corresponding unburned plots. Although absolute MNC values were generally higher in the topsoil than in the subsoil, the relative increase under moderate wildfire was larger in the subsoil.

**Figure 4:**
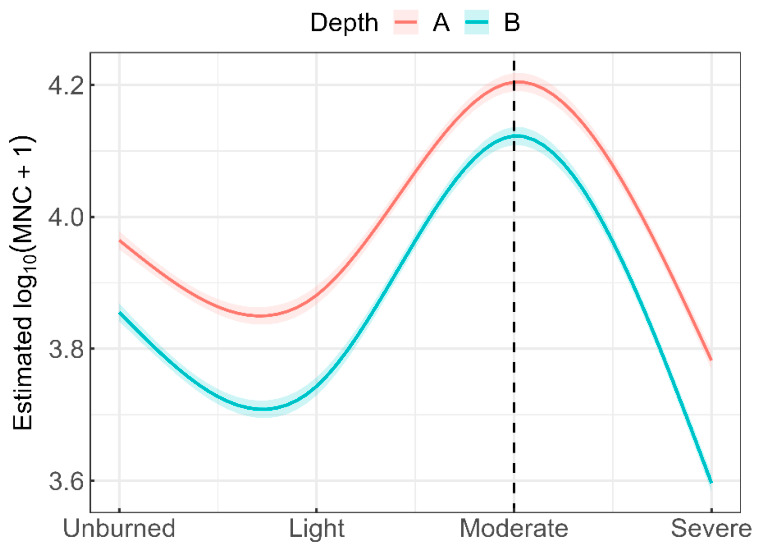
Generalized additive model fits showing changes in MNC along the wildfire-severity gradient. Lines indicate fitted values, and shaded areas indicate 95% confidence intervals. A and B indicate the 0–20 cm and 20–40 cm soil layers, respectively.

Generalized additive models further indicated a hump-shaped pattern of MNC along the wildfire-severity gradient in both soil layers, with fitted maxima under moderate wildfire and declines toward severe wildfire (Figure 4; Table S6). Because the study was conducted within a single wildfire-affected landscape, rather than across multiple independent wildfire events or replicated sites for each severity class, these patterns should be interpreted as observational variation along the wildfire-severity gradient rather than as independently replicated treatment effects.

### 3.2. Relationships Between Necromass Carbon and Soil Carbon Fractions

**Figure 5:**
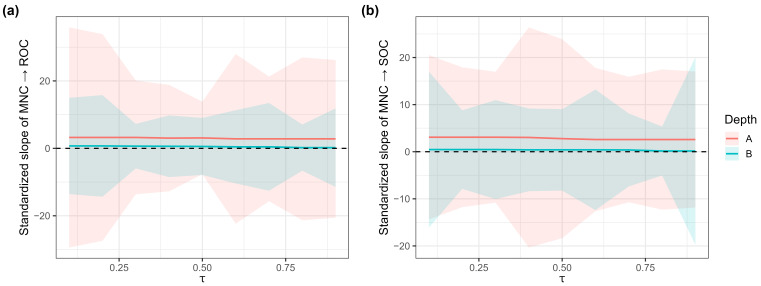
Depth-stratified quantile regression slopes of MNC for ROC and SOC. (**a**) Standardized quantile regression slopes of MNC for ROC. (**b**) Standardized quantile regression slopes of MNC for SOC. The dashed horizontal line indicates a zero slope, and shaded areas represent 95% confidence intervals. A and B indicate the 0–20 cm and 20–40 cm soil layers, respectively.

After accounting for soil physicochemical covariates and wildfire severity, the association between MNC and stable carbon pools differed clearly between soil layers (Table 2 and Table S7). In the topsoil layer, MNC was positively associated with both ROC (β = 3.644, 95% CI: 0.879–6.409, *p* = 0.018) and SOC (β = 3.436, 95% CI: 1.094–5.778, *p* = 0.012). The corresponding partial R^2^ values were high for ROC (0.6342) and SOC (0.6824), and nested model comparisons confirmed that adding MNC significantly improved model fit beyond the covariates alone. In contrast, no significant MNC–carbon pool associations were detected in the subsoil layer for either ROC (β = 0.495, 95% CI: −0.715 to 1.705, *p* = 0.356) or SOC (β = 0.308, 95% CI: −0.801 to 1.418, *p* = 0.522). Quantile regression and relative-importance analysis supported the same depth-dependent pattern, showing stronger MNC contributions to ROC and SOC in the topsoil than in the subsoil (Figure 5 and Figure S1). These results indicate that microbial necromass was more closely coupled with stable carbon pools in the topsoil layer.

### 3.3. Associations of Amino Sugars and MNC with Extracellular Enzyme Activities and CAZy Composite Indices

**Figure 6:**
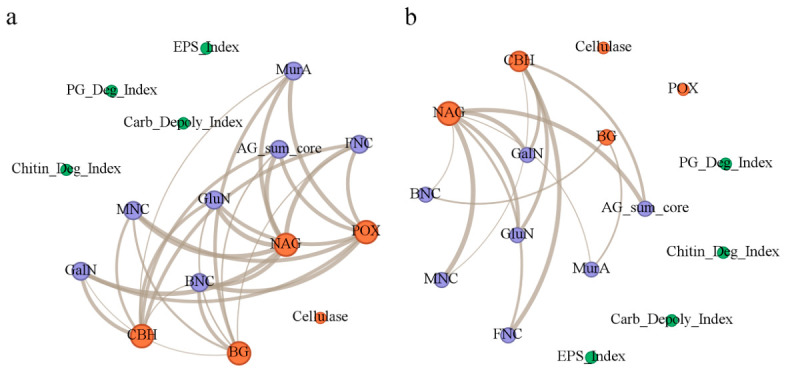
Partial-correlation networks among CAZy composite indices, extracellular enzyme activities, amino sugars, and microbial necromass carbon. Panels (**a**) and (**b**) represent the topsoil layer (0–20 cm) and subsoil layer (20–40 cm), respectively. Only edges meeting the thresholds of FDR-adjusted *p* < 0.05 and |r| > 0.30 were retained, and edge width is proportional to |r|. Node size is proportional to node degree, and node color indicates variable category. Nodes without edges indicate that their associations did not reach the significance threshold after FDR correction. All retained significant edges represent positive partial correlations.

Partial-correlation analysis showed that, after controlling for pH, BD, and SMC, significant associations were concentrated mainly between extracellular enzyme activities and amino sugars or necromass pools (Figure 6; Tables S10 and S11). By contrast, the CAZy composite indices did not show robust associations with amino sugars or necromass variables after FDR correction.

In the topsoil layer, NAG showed strong positive correlations with AG_sum_core, GluN, and MNC, with partial r values of 0.9978, 0.9977, and 0.9957, respectively (Table 3). In the subsoil layer, NAG remained strongly correlated with MNC (partial r = 0.9854, FDR-adjusted *p* = 2.89 × 10^−8^), and CBH was strongly correlated with FNC (partial r = 0.9708, FDR-adjusted *p* = 6.44 × 10^−7^). Overall, measured extracellular enzyme activities were more closely associated with amino sugars and microbial necromass than were the CAZy-based functional indices.

Overall, under the current dataset, extracellular enzyme activities showed more stable statistical associations with amino sugars and necromass pools than did the CAZy composite indices. This pattern suggests that measured enzyme activities better captured near-term process-related variation linked to amino sugars and microbial necromass, whereas the composite CAZy indices showed weaker correspondence with necromass-related carbon pools. Weak associations that did not survive FDR correction but remained visible in the exploratory networks are shown in Figure S2.

### 3.4. Exploratory Path Framework and Chained Associations

**Figure 7:**
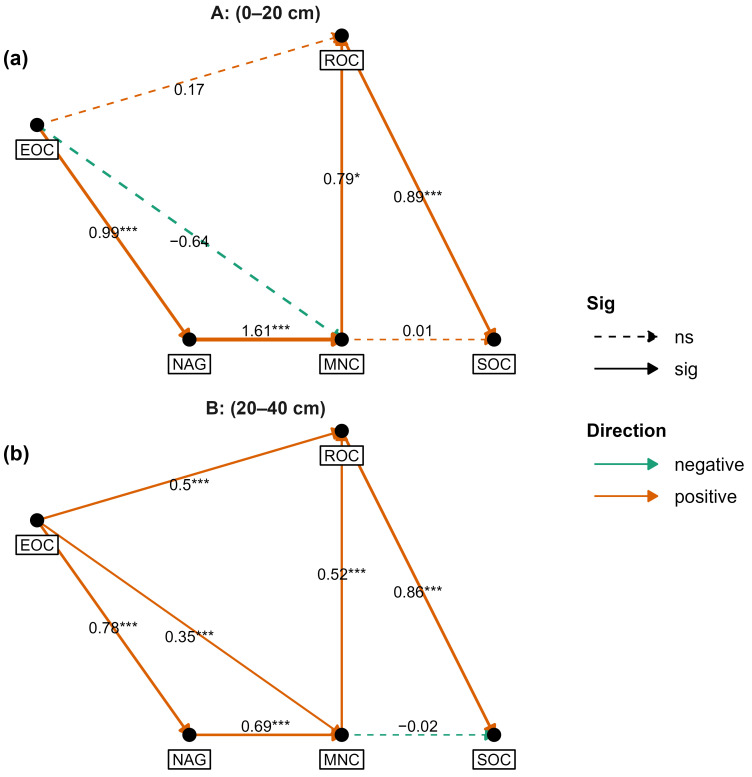
Depth-stratified EOC–NAG–MNC–ROC/SOC path models. (**a**) Path model for the topsoil layer (0–20 cm). (**b**) Path model for the subsoil layer (20–40 cm). Arrows indicate regression paths among variables, and values beside arrows denote standardized path coefficients (β). Line width is proportional to the absolute value of β. Solid and dashed arrows indicate significant and non-significant paths, respectively. Asterisks indicate significance levels (* *p* < 0.05, *** *p* < 0.001). All continuous variables were Z-standardized before model fitting. Path coefficients were derived from depth-stratified linear models (see Table 4).

The exploratory path analysis revealed distinct depth-dependent association structures among EOC, NAG, MNC, ROC, and SOC (Figure 7; Table 4). In the topsoil layer, the strongest positive paths followed the sequence EOC → NAG → MNC → ROC → SOC. Specifically, EOC was strongly associated with NAG (β = 0.99, *p* < 0.001), NAG was positively associated with MNC (β = 1.61, *p* < 0.001), MNC was positively associated with ROC (β = 0.79, *p* < 0.05), and ROC was strongly associated with SOC (β = 0.89, *p* < 0.001).

In the subsoil layer, the EOC → NAG and NAG → MNC paths remained significant but were weaker than those in the topsoil layer. Meanwhile, direct associations of EOC with MNC and ROC became more evident. Chained indirect effects also declined from the topsoil to the subsoil, with the full EOC → NAG → MNC → ROC → SOC pathway decreasing from 1.124 in the topsoil to 0.243 in the subsoil (Table S12). These results suggest that the necromass-mediated carbon stabilization pathway was more pronounced in the topsoil, whereas subsoil carbon variation reflected a more mixed set of direct and indirect associations.

## 4. Discussion

### 4.1. A Peak in Microbial Necromass Under Moderate Wildfire and Its Depth Dependence

In the present study, MNC reached its highest values in the moderate-wildfire class in both the topsoil layer and subsoil layer, whereas lower values were observed in the light- and severe-wildfire plots. Relative to the unburned plots, the proportional contrast associated with moderate wildfire was larger in the subsoil layer (20–40 cm) than in the topsoil layer (0–20 cm). These results indicate a clear peak in microbial necromass under moderate wildfire across the observed wildfire gradient and suggest that the contrast among wildfire severity classes differed between soil layers.

This non-linear response may reflect a balance between fire-induced substrate modification and fire-induced biological stress. Moderate wildfire may have maintained sufficient belowground and litter-derived inputs while modifying the quality of organic substrates and soil microenvironmental conditions. These conditions could favor microbial recovery, assimilation, and turnover, thereby promoting the formation of microbial residues and amino sugar accumulation. In contrast, severe wildfire may cause stronger combustion of the organic layer, greater damage to fine roots, and stronger disruption of microbial habitats, thereby reducing microbial biomass recovery and necromass production. This interpretation is consistent with previous studies showing that fire effects on soil carbon persistence depend on both changes in organic matter chemistry and environmental controls on decomposition [2], and that microbial necromass formation is closely linked to microbial biomass turnover and soil organic matter accumulation [5,6].

Importantly, however, this study was conducted within a single wildfire-affected landscape, rather than across multiple independent wildfire events or replicated sites for each severity class. In addition, background vegetation composition was not identical among the stands associated with different wildfire-severity classes. For example, the moderate wildfire plot was dominated by *Pinus massoniana*, the severe-wildfire plot by *Cryptomeria fortunei*, whereas the unburned and light wildfire plots were dominated by *Pinus armandii*. Accordingly, the observed “moderate-wildfire peak” should be interpreted as a pattern of variation among spatially separated sampling points distributed along a wildfire-severity gradient rather than as a strictly independent causal effect of wildfire severity itself. Even so, the direction of this pattern is broadly consistent with previous studies suggesting that low- to moderate-wildfire conditions, or longer post-fire recovery, may be associated with greater microbial necromass accumulation, whereas high burn intensity or repeated burning is more often associated with reduced necromass formation.

Global syntheses have shown that fire effects on soil carbon pools and microbial biomass are highly context dependent. High-severity wildfire and repeated burning often reduce SOC and microbial biomass, particularly in surface soils, whereas lower-intensity burning may show weaker negative effects and, under some conditions, little decline or partial recovery over time [1,2,3]. Within this broader context, the pattern observed here—higher MNC in plots under moderate wildfire and a larger proportional contrast at 20–40 cm—may reflect several non-exclusive processes. One possible explanation is that moderate wildfire conditions were associated with intermediate substrate inputs, altered hydrothermal conditions, and enhanced microbial turnover, all of which could favor necromass accumulation. By contrast, lower MNC in severe wildfire plots may reflect stronger damage to the organic layer, root systems, and microbial habitat, thereby reducing substrate availability and microbial recovery. The relatively larger proportional contrast in the subsoil layer may further suggest that post-fire changes in dissolved organic inputs, root turnover, or downward movement of necromass-related materials extended beyond the surface layer.

Overall, this study provides observational evidence that microbial necromass varied markedly along the wildfire gradient and that the contrast associated with moderate wildfire differed with soil depth. These findings do not demonstrate an independently replicated treatment effect, but they highlight a depth-dependent field pattern of greater necromass-related carbon accumulation under moderate wildfire conditions.

### 4.2. Coupling Between Microbial Necromass and Stable Carbon Pools

A key result of this study was that MNC showed much stronger statistical associations with ROC and SOC in the topsoil layer than in the subsoil layer. In the topsoil layer, MNC explained a relatively large proportion of the variation in both ROC and SOC after adjustment for soil physicochemical covariates and wildfire severity, whereas these associations weakened substantially and became non-significant in the subsoil layer. This pattern suggests closer coupling between microbial necromass and stable carbon pools in the topsoil layer and is consistent with the idea that necromass-related carbon processing may be more strongly expressed near the soil surface.

The stronger MNC–ROC/SOC association in the topsoil is ecologically plausible because surface soils usually receive greater inputs of litter-derived carbon, root exudates, and fine-root turnover than deeper soils. These inputs can stimulate microbial assimilation and turnover, thereby increasing the production of microbial residues. According to the microbial efficiency–matrix stabilization framework, labile plant-derived carbon can contribute to stable soil organic matter when it is efficiently assimilated by microorganisms and subsequently stabilized as microbial products through mineral association and aggregate protection [27]. Direct experimental evidence also shows that microbial-derived compounds can represent an important source of persistent soil organic matter [28]. Therefore, the stronger topsoil coupling observed in this study suggests that microbial necromass may act as an important intermediate pool linking post-fire microbial processing with relatively stable carbon fractions.

This depth contrast is broadly consistent with current understanding of microbial necromass as an important contributor to SOC formation. Cross-ecosystem syntheses indicate that microbial necromass commonly contributes a substantial fraction of SOC, especially in the topsoil layer, although the strength of this contribution varies with depth, mineral composition, and aggregate protection [5,6,29,30,31]. Compared with these broader patterns, the weaker associations observed in the subsoil layer may reflect several characteristics of the present study system. First, the 20–40 cm layer likely represents a transitional soil layer rather than a deeply stabilized mineral subsoil, and may still receive root-derived inputs and dissolved organic matter. Second, ROC was operationally calculated as SOC minus EOC; therefore, it should be interpreted as a relatively stable carbon fraction rather than as a direct measure of mineral-associated or microbial-derived carbon. This fraction may include multiple carbon sources, including microbial residues, mineral-associated organic matter, and fire-derived pyrogenic carbon. Third, soils were sampled approximately four months after the wildfire, and the contribution of microbial necromass to more persistent carbon fractions may not yet have been fully expressed over this short post-fire interval. Therefore, the weaker subsoil MNC–ROC/SOC associations do not necessarily indicate that microbial necromass is unimportant at depth, but rather suggest that subsoil carbon stabilization is controlled by a broader set of biological and abiotic processes [2,27].

Even after accounting for pH, moisture, bulk density, DOC, MBC, and wildfire severity class, MNC still contributed substantially to fitted models of ROC and SOC in the topsoil layer. Under the present observational framework, this result supports the view that microbial necromass remained an important associated factor in stable carbon variation, especially near the soil surface. At the same time, the weaker subsoil relationships suggest that stable carbon in the subsoil layer reflected a broader combination of post-fire inputs and stabilization pathways rather than a single dominant necromass-related association.

### 4.3. Differences Between Enzyme Activities and CAZy Composite Indices in Their Associations with Amino Sugars and Microbial Necromass

The partial-correlation analyses showed that, after accounting for pH, BD, and SMC, the most stable associations retained after FDR correction were concentrated between extracellular enzyme activities and amino sugars or necromass pools, whereas the CAZy composite indices did not retain robust associations with necromass-related variables in either the topsoil layer or the subsoil layer. In both the topsoil layer and subsoil layer, NAG, BG, and CBH showed strong positive correlations with amino sugars and microbial necromass, whereas the composite CAZy indices showed much weaker and less consistent relationships. Under the present dataset, this pattern suggests that measured extracellular enzyme activities were more closely aligned with variation in amino sugars and necromass pools than were the CAZy-based functional indices.

Among the measured extracellular enzymes, NAG showed particularly strong associations with amino sugars and MNC. This relationship is mechanistically meaningful because NAG participates in the degradation of N-acetylglucosamine-containing polymers such as chitin and peptidoglycan, which are major structural components of fungal and bacterial cell walls. Amino sugars, especially glucosamine and muramic acid, are widely used as biomarkers of fungal and bacterial residues in soil [8]. Therefore, the strong NAG–MNC association supports the interpretation that microbial cell-wall turnover was closely linked to necromass accumulation in the studied post-fire soils.

The contrast between extracellular enzyme activities and CAZy composite indices is important because they represent different levels of microbial function. Extracellular enzyme activities reflect realized biochemical processes occurring under the current soil environment, whereas metagenome-derived CAZy family abundances represent the functional potential of microbial communities to produce enzymes involved in the synthesis, modification, and degradation of carbohydrates [32]. Therefore, CAZy family abundance may not translate directly into measured enzyme activity or microbial necromass accumulation, especially during the early post-fire stage when substrate availability, microbial physiological state, and soil microenvironmental conditions may change rapidly. This may explain why extracellular enzyme activities showed stronger correlations with amino sugars and MNC, whereas the CAZy composite indices did not retain robust associations after FDR correction. This interpretation is also consistent with the view that microbial functional gene abundance and extracellular enzyme activity are related but not equivalent indicators of soil carbon cycling processes [11].

Overall, the present results suggest that extracellular enzyme activities captured near-term process-related variation more directly than the composite CAZy indices, whereas the latter may better represent background functional potential. This distinction is important for interpreting post-fire soil carbon stabilization, because it indicates that gene-level functional information and measured enzyme activity should not be assumed to track necromass-related carbon pools in the same way.

### 4.4. Depth-Dependent Differences in the Necromass-Related EOC-ROC Association Framework

The exploratory path analyses indicated that the standardized association framework linking EOC, NAG, MNC, ROC, and SOC differed clearly between the topsoil layer and subsoil layer. In the topsoil layer, the strongest positive paths followed the sequence EOC → NAG → MNC → ROC → SOC, whereas direct EOC–MNC and EOC–ROC paths were comparatively weak or non-significant. This pattern is consistent with a more concentrated chained association structure in the topsoil layer, in which EOC-related variation was linked to ROC and SOC primarily through enzyme activity and microbial necromass.

From a mechanistic perspective, this pathway is consistent with the concept that labile carbon can contribute to more persistent soil carbon pools after microbial assimilation and transformation. EOC represents a relatively active and disturbance-sensitive carbon fraction that can serve as a potential substrate pool for microbial processing. Enhanced NAG activity may indicate intensified microbial processing of nitrogen-containing microbial or fungal residues, while the subsequent positive MNC–ROC association suggests that part of this microbial-derived material may become incorporated into relatively stable carbon pools. This interpretation is consistent with the view that microbial transformation and mineral or aggregate protection jointly regulate the formation of persistent soil organic matter [5,27,28].

By contrast, in the subsoil layer, the positive EOC–NAG and NAG–MNC paths remained but were weaker, while direct EOC–MNC and EOC–ROC paths became more evident. Together with the positive MNC–ROC path, this suggests a more mixed framework in the subsoil layer, in which EOC-related variation was linked to stable carbon pools through both chained and more direct associations. The smaller chained indirect effects in the subsoil layer further support this interpretation.

Conceptually, this depth contrast is consistent with the broader view that labile carbon inputs may be processed through microbial pathways more strongly in the topsoil layer, whereas carbon variation in the subsoil layer may reflect a combination of microbial transformation, dissolved organic inputs, mineral interactions, and fire-derived carbon fractions. However, because the present path analyses were based on standardized linear equations fitted separately within each layer, and because no overall goodness-of-fit test was performed, these results should be interpreted as relative comparisons of association structure rather than as confirmation of a definitive mechanistic sequence.

The present findings therefore suggest that post-fire carbon stabilization may differ with depth not only in magnitude but also in the dominant configuration of associated pathways. In the topsoil layer, the EOC–NAG–MNC–ROC–SOC chain showed stronger support as an exploratory association framework, whereas in the subsoil layer, stable carbon variation appeared to reflect a more mixed set of direct and necromass-related associations.

However, this framework should be interpreted cautiously. The path analysis was based on observational data and standardized linear associations rather than experimental manipulation or isotope tracing. Therefore, the identified EOC–NAG–MNC–ROC/SOC pathway should be viewed as a hypothesis-generating association framework rather than direct evidence of causal carbon transfer. Future studies using isotope-labeled substrates, microbial biomarker tracing, and physical fractionation of mineral-associated organic carbon will be needed to directly test whether microbial necromass is incorporated into persistent carbon pools after wildfire.

### 4.5. Limitations and Implications

Several limitations of this study should be acknowledged. First, this study was conducted within a single wildfire-affected landscape, rather than across multiple independent wildfire events or replicated sites for each wildfire-severity class. Therefore, the results should be interpreted as observational patterns along a wildfire-severity gradient rather than as independently replicated treatment effects. Second, soil samples were collected approximately four months after the wildfire, so the results reflect early post-fire conditions rather than long-term recovery trajectories. Third, vegetation composition differed among the stands associated with different wildfire-severity classes, which may have influenced litter quality, root inputs, microbial communities, and soil carbon dynamics. Finally, ROC was operationally calculated as SOC minus EOC, and this fraction may include multiple carbon sources, including microbial necromass-derived carbon and pyrogenic carbon.

Despite these limitations, this study provides useful evidence that microbial necromass is associated with depth-dependent post-fire carbon stabilization. The stronger coupling between MNC and ROC/SOC in the topsoil highlights the potential importance of microbial residues in surface soil carbon stabilization after wildfire. Future studies combining replicated wildfire gradients, longer post-fire chronosequences, deeper soil profiles, mineral-associated organic carbon measurements, and isotope tracing would help clarify the causal role of microbial necromass in post-fire soil carbon persistence.

## 5. Conclusions

Based on observations within a single wildfire-affected landscape in a subtropical coniferous forest, this study showed that microbial necromass accumulation and its associations with stable carbon pools varied across wildfire severity classes and soil depths. The moderate-wildfire class exhibited the highest MNC values in both the topsoil layer and subsoil layer, and MNC showed stronger statistical coupling with ROC and SOC in the topsoil layer. In contrast, the subsoil layer displayed a more mixed pattern, in which both necromass-related associations and more direct EOC-related associations were linked to variation in stable carbon pools. Extracellular enzyme activities also showed closer associations with amino sugars and necromass pools than did the composite CAZy functional indices.

Overall, these results suggest a depth-dependent pattern in post-fire soil carbon stabilization, with stronger necromass-related coupling in the topsoil layer and a comparatively more complex association framework in the subsoil layer. Because the study was conducted within a single wildfire-affected landscape rather than across multiple independent wildfire events or replicated sites for each wildfire-severity class, the findings should be interpreted as observational evidence rather than as independently replicated treatment effects. Future studies incorporating replicated wildfire gradients, longer post-fire chronosequences, and deeper soil profiles will be needed to further clarify the role of microbial necromass in post-fire carbon stabilization.

## Figures and Tables

**Table 1:** Characteristics of wildfire-severity plots.

Wildfire Severity	Latitude and Longitude Range	Representative Altitude (m)	Representative Slope Direction	Representative Slope (°)	Vegetation Types	Soil Types	Soil Texture	Fire Type	Burned Wood	Flame Height (m)	Vegetation Damage	Soil-Surface Condition	Wildfire Occurrence	Sampling Time
Unburned (CK)	106°30′29.15″ E–106°30′35.39″ E 26°15′58.40″ N–26°16′04.58″ N	1419.46	N	3.5	*Pinus armandii*	Acrisols	Loam	/	/	/	/	/	/	/
Light wildfire (L)	106°30′38.53″ E–106°30′42.09″ E 26°16′40.98″ N–26°16′43.45″ N	1429.06	SE	10.5	*Pinus armandii*	Acrisols	Loam	Surface fire	≤30%	<1.5	The trunk is scorched, and the tree is still covered with green leaves	The soil organic matter layer is intact, and the carbonization depth is only a few millimeters	February 2024	4 months post-fire
Moderate wildfire (M)	106°30′53.56″ E–106°31′03.84″ E 26°16′58.55″ N–26°16′59.79″ N	1358.90	E	10.6	*Pinus massoniana*	Acrisols	Loam	Surface fire	30–70%	1.5–3.0				
Severe wildfire (S)	106°30′37.69″ E–106°30′40.86″ E 26°16′45.53″ N–26°16′47.13″ N	1317.60	NW	11.9	*Cryptomeria fortunei*	Acrisols	Loam	Surface fire Crown fire	≥70%	>3.0	The crown was burned, and no green leaves covered it.	Ash deposits and charred organic matter are a few centimeters thick		

Note: Reference basis for classification of wildfire severity [13,14].

**Table 2:** Depth-stratified associations of MNC with ROC and SOC.

Response	β _A (95% CI)	β_B (95% CI)	Partial R^2^_A	Partial R^2^_B	ΔR^2^_MNC (Overall)	Partial R^2^ (Overall)	*n*_A	*n*_B
ROC_z	3.644 [0.879, 6.409]	0.495 [−0.715, 1.705]	0.6342	0.1431	3.88 × 10^−4^	0.0817	16	16
SOC_z	3.436 [1.094, 5.778]	0.308 [−0.801, 1.418]	0.6824	0.0715	1.15 × 10^−4^	0.0319	16	16

Note: β_A and β_B represent standardized slopes for the topsoil layer and subsoil layer, respectively. Partial R^2^ indicates the independent explanatory contribution of MNC after controlling for covariates. A = 0–20 cm; B = 20–40 cm.

**Table 3:** Major FDR-corrected partial correlations between extracellular enzyme activities and amino sugars/MNC.

Depth	Predictor Type	Predictor	Response	Partial r	*p* Value	FDR-Adjusted *p*	*n*
A	Enzyme	NAG	AG_sum_core	0.9978	2.31 × 10^−14^	5.16 × 10 ^−13^	16
	Enzyme	NAG	GluN	0.9977	2.95 × 10^−14^	5.16 × 10 ^−13^	16
	Enzyme	NAG	MNC	0.9957	9.89 × 10^−13^	1.15 × 10 ^−11^	16
B	Enzyme	NAG	MNC	0.9854	8.27 × 10^−10^	2.89 × 10 ^−8^	16
	Enzyme	CBH	FNC	0.9708	3.68 × 10^−8^	6.44 × 10 ^−7^	16
	Enzyme	NAG	AG_sum_core	0.9630	1.33 × 10^−7^	1.55 × 10 ^−6^	16

Note: A = 0–20 cm; B = 20–40 cm. NAG and CBH represent N-acetylglucosaminidase and cellobiohydrolase. GluN represents glucosamine. MNC and FNC represent microbial and fungal necromass carbon, respectively. AG_sum_core represents the sum of core amino sugars. *n* represents the sample size.

**Table 4:** Standardized regression coefficients for the chained EOC–NAG–MNC–ROC/SOC pathways across soil layers.

Depth	Response	Predictor	Standardized Coefficient, β	SE	*p* Value	R^2^	*n*
A	NAG	EOC	0.99	0.04	3.79 × 10^−13^	0.98	16
	MNC	EOC	−0.64	0.3	0.0519	0.98	16
	ROC	EOC	0.17	0.27	0.5400	0.92	16
	MNC	NAG	1.61	0.3	1.18 × 10^−4^	0.98	16
	ROC	MNC	0.79	0.27	0.0125	0.92	16
	SOC	MNC	0.01	0.02	0.5990	1.00	16
	SOC	ROC	0.89	0.02	9.53 × 10^−15^	1.00	16
B	NAG	EOC	0.78	0.17	3.27 × 10^−4^	0.61	16
	MNC	EOC	0.35	0.04	1.12 × 10^−6^	0.99	16
	ROC	EOC	0.50	0.06	1.40 × 10^−6^	0.99	16
	MNC	NAG	0.69	0.04	3.75 × 10^−10^	0.99	16
	ROC	MNC	0.52	0.06	9.31 × 10^−7^	0.99	16
	SOC	MNC	−0.02	0.02	0.4090	1.00	16
	SOC	ROC	0.86	0.04	6.53 × 10^−11^	1.00	16

Note: All continuous variables were Z-standardized before model fitting. A = 0–20 cm; B = 20–40 cm. β indicates standardized path coefficient.

## Data Availability

The raw metagenomic sequencing data have been deposited in the NCBI Sequence Read Archive (SRA) under BioProject accession number PRJNA1280738 and SRA accession number SRP593774.

## Supplementary Materials

The following supporting information can be downloaded at: https://www.mdpi.com/article/10.3390/microorganisms14061380/s1, Figure S1: Stratified relative importance of explanatory variables for ROC and SOC based on the lmg metric; Figure S2: Exploratory partial-correlation networks among composite functional indices, extracellular enzyme activities, and amino sugars/microbial necromass carbon across soil layers; Table S1: Soil physicochemical properties across wildfire-severity classes and soil depths; Table S2: Soil carbon fractions across wildfire-severity classes and soil depths; Table S3: Sequencing depth and metagenomic assembly statistics for each soil sample; Table S4: Type III ANOVA for the effects of wildfire severity, soil depth, and their interaction on log_10_(MNC + 1); Table S5: EMMs of MNC for each Wildfire × Depth combination; Table S6: Significance of GAM smooth terms by soil layer; Table S7: Stratified partial R^2^ and nested F-tests for the effects of MNC on ROC and SOC; Table S8: Correspondence between CAZy families, functional groupings, and composite functional indices; Table S9: Composite CAZy functional indices for each soil sample; Table S10: Partial-correlation matrix between composite functional indices and amino sug-ars/microbial necromass carbon stratified by soil layer, including FDR correction; Table S11: Partial-correlation matrix between enzyme activities and amino sugars/microbial necromass carbon stratified by soil layer, including FDR correction; Table S12: Chained indirect effects of EOC on NAG–MNC–ROC/SOC pathways.

## Abbreviations

The following abbreviations are used in this manuscript:

SOC	Soil organic carbon
EOC	Easily oxidizable organic carbon
ROC	Recalcitrant organic carbon
DOC	Dissolved organic carbon
MBC	Microbial biomass carbon
MNC	Microbial necromass carbon
FNC	Fungal necromass carbon
BNC	Bacterial necromass carbon
GluN	Glucosamine
GalN	Galactosamine
MurA	Muramic acid
CAZy	Carbohydrate-active enzyme database
CAZymes	Carbohydrate-active enzymes
BG	β-glucosidase
NAG	N-acetylglucosaminidase
CBH	Cellobiohydrolase
POX	Polyphenol oxidase
SMC	Soil moisture content
BD	Bulk density
STN	Soil total nitrogen
STP	Soil total phosphorus
STK	Soil total potassium
SAN	Soil alkali-hydrolyzable nitrogen
SAP	Soil available phosphorus
SAK	Soil available potassium
NO_3_^−^-N	Nitrate nitrogen
NH_4_^+^-N	Ammonium nitrogen
CK	Unburned control
L	Light wildfire
M	Moderate wildfire
S	Severe wildfire
