# Supplementary material for: Microbial Necromass and Extracellular Enzyme Activities Are Associated with Depth-Dependent Soil Carbon Stabilization Along a Wildfire-Severity Gradient
Source: Microorganisms. 2026 Jun 22;14(6):1380. doi: 10.3390/microorganisms14061380 (PMC13306029; doi:10.3390/microorganisms14061380)
Supplement: Supplementary file 1 [file microorganisms-14-01380-s001.zip › microorganisms-4397821-supplementary.pdf]

Supplementary Materials

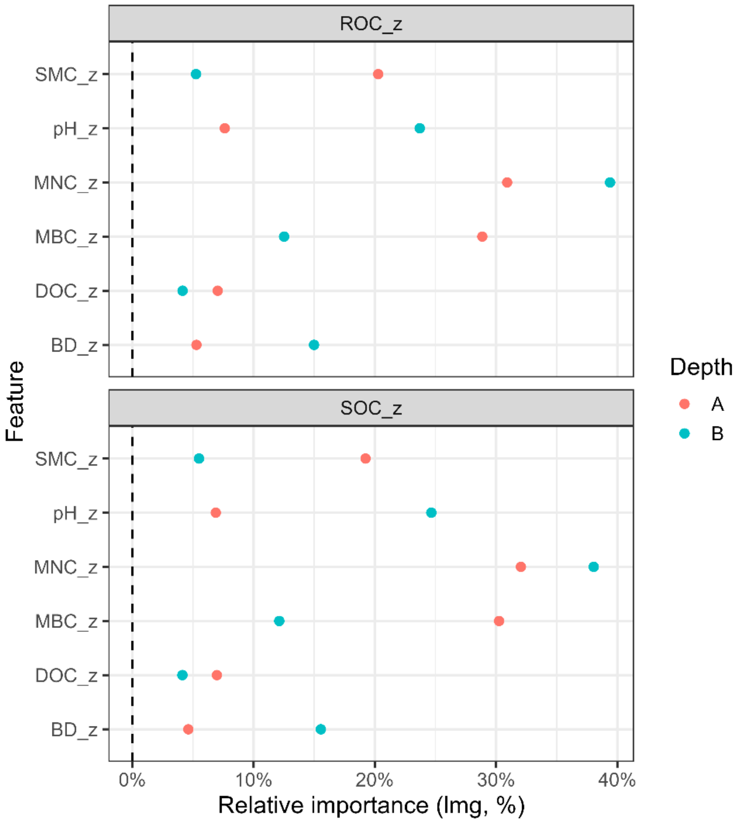

Figure S1. Stratified relative importance of explanatory variables for ROC and SOC based on the lmg metric. Relative importance was calculated using the lmg metric. A = topsoil layer (0–20 cm); B = subsoil layer (20–40 cm).

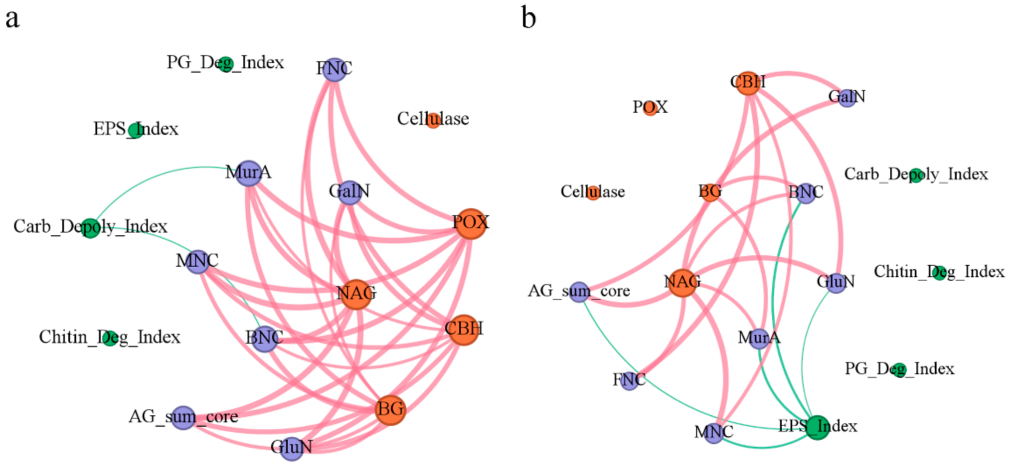

Figure S2. Exploratory partial-correlation networks among composite functional indices, extracellular enzyme activities, and amino sugars/microbial necromass carbon across soil layers. Panels (a) and (b) represent the topsoil layer (0–20 cm) and subsoil layer (20–40 cm), respectively. Only associations with $P < 0.05$ and $|r| > 0.30$ are shown.

Table S1. Soil physicochemical properties across wildfire-severity classes and soil depths

Soil layers	Wildfire	SMC (%)	BD ($g\cdot cm^{-3}$)	pH	STN ($g\cdot kg^{-1}$)	STP ($g\cdot kg^{-1}$)	STK ($g\cdot kg^{-1}$)	SAN ($mg\cdot kg^{-1}$)	SAP ($mg\cdot kg^{-1}$)	SAK ($mg\cdot kg^{-1}$)	NO_3^--N ($\mu g\cdot g^{-1}$)	NH_4^+-N ($\mu g\cdot g^{-1}$)
A: 0–20 cm	CK	22.50±0.52 Ad	1.22±0.01 Aa	4.42±0.00 Aa	1.67±0.03 Ac	0.27±0.00 Ac	2.03±0.07 Bd	81.67±0.82 Ad	29.13±0.13 Aa	27.79±2.24 Ac	2.42±0.08 Bc	25.16±0.13 Ad
	Light	31.39±0.74 Ab	0.94±0.06 Ab	4.13±0.00 Bd	1.40±0.00 Ad	0.56±0.00 Aa	5.66±0.08 Ba	95.67±1.65 Ac	19.60±0.30 Ac	68.99±0.00 Ab	4.07±0.06 Ab	38.27±0.75 Aa
	Moderate	38.62±0.43 Aa	0.87±0.08 Ab	4.15±0.00 Bc	2.76±0.03 Aa	0.50±0.00 Ab	3.11±0.04 Ac	179.67±1.65 Aa	21.31±0.08 Ab	78.50±0.00 Aab	4.37±0.17 Ab	30.14±0.42 Ac
	Severe	27.94±0.43 Ac	1.02±0.05 Ab	4.34±0.00 Ab	1.79±0.06 Ab	0.51±0.01 Ab	4.58±0.04 Bb	101.50±1.43 Ab	21.42±0.13 Ab	94.34±4.48 Aa	7.51±0.08 Aa	33.78±0.65 Ab
B: 20–40 cm	CK	24.08±0.71 Ac	1.22±0.02 Aa	4.32±0.00 Ba	0.78±0.03 Bb	0.19±0.00 Bc	3.00±0.07 Ac	56.00±1.43 Bc	17.35±0.15 Bb	15.12±2.24 Bb	3.62±0.06 Ab	24.63±0.12 Ac
	Light	31.76±0.25 Aa	1.06±0.01 Ab	4.22±0.00 Ac	0.78±0.03 Bb	0.55±0.00 Aa	6.36±0.08 Aa	57.17±0.82 Bbc	17.03±0.08 Bb	40.47±3.88 Ba	3.10±0.06 Bc	29.25±0.55 Ba
	Moderate	29.31±0.48 Bb	0.86±0.06 Ac	4.19±0.00 Ad	2.65±0.05 Aa	0.44±0.01 Bb	2.30±0.04 Bd	121.33±0.82 Ba	20.99±0.08 Aa	37.30±2.24 Ba	4.14±0.20 Aa	28.84±0.09 Ba
	Severe	27.53±1.20 Ab	1.06±0.04 Ab	4.29±0.00 Bb	0.74±0.03 Bb	0.44±0.01 Bb	5.49±0.04 Ab	59.50±1.43 Bb	16.49±0.15 Bc	37.30±2.24 Ba	3.29±0.02 Bc	27.01±0.56 Bb

Note: Values are mean ± SE. Lowercase letters (a–d) indicate significant differences among wildfire-severity classes within the same soil layer, whereas uppercase letters (A–C) indicate significant differences between soil layers within the same wildfire-severity class (emmeans, BH-FDR-adjusted $P < 0.05$).

Table S2. Soil carbon fractions across wildfire-severity classes and soil depths.

Soil layers	Wildfire severity	SOC ($g·kg^{-1}$)	DOC ($mg·kg^{-1}$)	MBC ($mg·kg^{-1}$)	EOC ($g·kg^{-1}$)	ROC ($g·kg^{-1}$)
A 0–20 cm	CK	42.27±0.98 Ab	107.92±0.31 Bb	187.39±0.12 Ab	9.24±0.09 Ab	32.74±0.89 Ab
	Light	38.29±0.56 Ac	127.70±0.09 Aa	50.75±0.48 Ad	6.93±0.13 Ac	31.18±0.44 Ab
	Moderate	82.15±1.13 Aa	31.98±0.25 Ac	386.78±0.49 Aa	12.84±0.09 Aa	68.89±1.05 Aa
	Severe	39.08±0.56 Abc	21.27±0.12 Bd	160.62±0.10 Ac	7.54±0.18 Ac	31.36±0.50 Ab
B 20–40 cm	CK	19.94±0.57 Bc	113.18±0.26 Aa	106.07±0.37 Bc	2.96±0.09 Bc	16.76±0.50 Bb
	Light	23.93±0.56 Bb	26.63±0.17 Bc	12.53±0.13 Bd	5.34±0.35 Bb	18.55±0.48 Bb
	Moderate	57.43±0.56 Ba	32.30±0.31 Ab	199.70±0.23 Ba	11.35±0.12 Ba	45.84±0.49 Ba
	Severe	13.56±0.98 Bd	25.78±0.08 Ad	136.51±0.15 Bb	2.99±0.07 Bc	10.41±0.90 Bc

Note: Values are mean ± SE. Lowercase letters (a–d) indicate significant differences among wildfire-severity classes within the same soil layer, whereas uppercase letters (A–C) indicate significant differences between soil layers within the same wildfire-severity class (emmeans, BH-FDR-adjusted P < 0.05).

Table S3. Sequencing depth and metagenomic assembly statistics for each soil sample.

Sample ID	Raw reads	Raw base (bp)	Clean base (bp)	Contigs	Contigs bases (bp)	N50 (bp)
CK-A-1	43,977,772	6,640,643,572	6,566,374,199	685,643	366,237,481	525
CK-A-2	42,792,140	6,461,613,140	6,390,211,780	728,078	430,645,455	587
CK-A-3	42,763,584	6,457,301,184	6,388,599,124	677,514	413,814,988	612
CK-A-4	52,829,478	7,977,251,178	7,823,061,139	689,372	388,766,251	555
CK-B-1	46,387,766	7,004,552,666	6,931,294,470	895,088	509,808,006	559
CK-B-2	41,067,020	6,201,120,020	6,136,232,014	710,213	427,927,053	597
CK-B-3	43,535,140	6,573,806,140	6,499,081,299	716,236	416,247,905	573
CK-B-4	50,868,772	7,681,184,572	7,543,438,703	727,733	461,161,485	631
Light-A-1	42,368,612	6,397,660,412	6,322,374,603	634,236	353,672,483	543
Light-A-2	47,711,322	7,204,409,622	7,120,340,613	816,618	459,388,558	545
Light-A-3	47,099,326	7,111,998,226	7,032,173,290	789,336	494,483,658	627
Light-A-4	52,735,038	7,962,990,738	7,805,376,210	734,880	512,750,378	735
Light-B-1	43,087,258	6,506,175,958	6,432,387,352	740,366	460,437,504	623
Light-B-2	43,382,324	6,550,730,924	6,480,293,165	705,165	467,238,874	687
Light-B-3	41,916,668	6,329,416,868	6,254,418,778	608,771	384,538,990	635
Light-B-4	49,655,196	7,497,934,596	7,352,834,260	854,806	567,402,101	698
Moderate-A-1	52,412,966	7,914,357,866	7,744,917,104	576,418	306,916,493	523
Moderate-A-2	51,188,048	7,729,395,248	7,513,201,081	695,050	375,014,376	519
Moderate-A-3	56,165,508	8,480,991,708	8,306,188,047	726,044	430,565,145	593
Moderate-A-4	51,298,756	7,746,112,156	7,595,621,454	627,256	338,424,393	525
Moderate-B-1	50,869,026	7,681,222,926	7,508,635,020	653,398	382,931,434	587
Moderate-B-2	51,548,842	7,783,875,142	7,626,196,426	647,445	352,185,849	533
Moderate-B-3	52,418,724	7,915,227,324	7,743,053,779	609,058	326,979,599	529
Moderate-B-4	46,602,678	7,037,004,378	6,912,949,094	702,866	399,561,167	559
Severe-A-1	45,996,118	6,945,413,818	6,787,341,616	567,352	327,631,166	571
Severe-A-2	49,525,184	7,478,302,784	7,319,586,260	645,570	385,415,517	595
Severe-A-3	51,006,014	7,701,908,114	7,539,074,548	646,881	368,036,650	566
Severe-A-4	50,599,390	7,640,507,890	7,505,946,554	669,324	411,179,337	609
Severe-B-1	50,933,400	7,690,943,400	7,543,517,534	759,135	474,421,249	631
Severe-B-2	54,371,050	8,210,028,550	8,058,251,078	924,627	588,253,816	658
Severe-B-3	51,001,436	7,701,216,836	7,561,011,837	724,262	480,190,723	691
Severe-B-4	50,347,842	7,602,524,142	7,446,026,237	810,251	535,475,744	691

Table S4. Type III ANOVA for the effects of wildfire severity, soil depth, and their interaction on $\log_{10}$(MNC + 1).

Effect	F	Df1	Df2	P	partial η^2	95% CI (partial η^2)
Wildfire	1629.146	3	24	7.452×10^{-28}	0.995	[0.992, 1.000]
Depth	665.596	1	24	5.174×10^{-19}	0.965	[0.940, 1.000]
Wildfire×Depth	19.889	3	24	1.072×10^{-6}	0.713	[0.514, 1.000]

Note: Partial η^2 is reported as the effect size, with its 95% confidence interval given in parentheses.

Table S5. EMMs of MNC for each Wildfire × Depth combination.

Depth	Wildfire Severity	MNC mean (mg C·kg^{-1})	95% CI lower (mg C·kg^{-1})	95% CI upper (mg C·kg^{-1})	n	% change	LCI%	UCI%
A	Unburned	9215.58 ^{Ab}	8911.72	9529.80	4	0	0	0
	Light	7602.01 ^{Ac}	7351.35	7861.22	4	−17.51%	−22.24%	−12.48%
	Moderate	16012.89 ^{Aa}	15484.93	16558.85	4	73.75%	63.78%	84.33%
	Severe	6054.79 ^{Ad}	5855.14	6261.25	4	−34.29%	−38.07%	−30.29%
B	Unburned	7165.80 ^{Bb}	6929.52	7410.14	4	0	0	0
	Light	5532.66 ^{Bc}	5350.22	5721.31	4	−22.79%	−27.22%	−18.09%
	Moderate	13266.63 ^{Ba}	12829.21	13718.96	4	85.13%	74.50%	96.40%
	Severe	3946.55 ^{Bd}	3816.40	4081.13	4	−44.92%	−48.08%	−41.56%

Note: Values are back-transformed estimated marginal means with 95% confidence intervals. Percentage changes were calculated relative to the unburned plots within the same soil layer. Uppercase letters indicate differences between soil layers within the same wildfire-severity class, whereas lowercase letters indicate differences among wildfire-severity classes within the same soil layer.

Table S6. Significance of GAM smooth terms by soil layer.

Depth	Smooth term	edf	Ref.df	F	p-value	Nonlinearity
A	s(Severity):byA	2.998	3.000	653.104	<1e-16	Yes ***
B	s(Severity):byB	2.999	3.000	993.830	<1e-16	Yes ***

Note: A = topsoil layer (0–20 cm); B = subsoil layer (20–40 cm). *** indicates P < 0.001.

Table S7. Stratified partial R^2 and nested F-tests for the effects of MNC on ROC and SOC.

Response	Depth	n	R^2_base	R^2_full	ΔR^2_MNC	partial_R^2	P (nested_F)	β_MNC	95% CI [LCI, UCI]	P	Sig
ROC	A	16	0.9961	0.9986	0.0025	0.6342	0.0180	3.6440	[0.879, 6.409]	0.018	*
ROC	B	16	0.9979	0.9982	0.0003	0.1431	0.3555	0.4951	[−0.715, 1.705]	0.356	ns
SOC	A	16	0.9965	0.9989	0.0024	0.6824	0.0115	3.4360	[1.094, 5.778]	0.012	*
SOC	B	16	0.9985	0.9986	0.0001	0.0715	0.5220	0.3082	[−0.801, 1.418]	0.522	ns

Note: Models were fitted separately for each soil layer. Partial R^2 indicates the independent explanatory contribution of MNC after controlling for covariates and wildfire severity. A = topsoil layer (0–20 cm); B = subsoil layer (20–40 cm).

Table S8. Correspondence between CAZy families, functional groupings, and composite functional indices.

Family	Group	Index	Family	Group	Index
GH18	chitinase	Chitin_Deg_Index	GH48	cellulose	Carb_Depoly_Index
GH19	chitinase	Chitin_Deg_Index	AA9	cellulose_LPMO	Carb_Depoly_Index
GH20	beta_hexosaminidase	Chitin_Deg_Index	AA10	cellulose_LPMO	Carb_Depoly_Index
GH46	chitosanase	Chitin_Deg_Index	GH10	hemicellulose	Carb_Depoly_Index
GH75	chitosanase	Chitin_Deg_Index	GH11	hemicellulose	Carb_Depoly_Index
GH80	chitosanase	Chitin_Deg_Index	GH12	hemicellulose	Carb_Depoly_Index
CE4	chitin_deacetylase	Chitin_Deg_Index	GH26	hemicellulose	Carb_Depoly_Index
AA10	LPMO_chitin	Chitin_Deg_Index	GH39	hemicellulose	Carb_Depoly_Index
GH22	lysozyme	PG_Deg_Index	GH43	hemicellulose	Carb_Depoly_Index
GH23	lytic_transgly	PG_Deg_Index	GH51	hemicellulose	Carb_Depoly_Index
GH24	lysozyme	PG_Deg_Index	GH53	hemicellulose	Carb_Depoly_Index
GH25	lysozyme	PG_Deg_Index	GH62	hemicellulose	Carb_Depoly_Index
GH73	endo_NAG	PG_Deg_Index	GH67	hemicellulose	Carb_Depoly_Index
GT2	EPS_GT	EPS_Index	GH115	hemicellulose	Carb_Depoly_Index
GT4	EPS_GT	EPS_Index	GH28	pectin	Carb_Depoly_Index
GT8	EPS_GT	EPS_Index	GH88	pectin	Carb_Depoly_Index
GT9	EPS_GT	EPS_Index	GH105	pectin	Carb_Depoly_Index
GT32	EPS_GT	EPS_Index	PL1	pectin_lyase	Carb_Depoly_Index
GT41	EPS_GT	EPS_Index	PL9	pectin_lyase	Carb_Depoly_Index
GT83	EPS_GT	EPS_Index	PL10	pectin_lyase	Carb_Depoly_Index
GH5	cellulose	Carb_Depoly_Index	PL11	pectin_lyase	Carb_Depoly_Index
GH6	cellulose	Carb_Depoly_Index	GH13	starch	Carb_Depoly_Index

GH7	cellulose	Carb_Depoly_Index	GH14	starch	Carb_Depoly_Index
GH9	cellulose	Carb_Depoly_Index	GH57	starch	Carb_Depoly_Index
GH44	cellulose	Carb_Depoly_Index	GH77	starch	Carb_Depoly_Index

Note: CAZy families were assigned to four composite indices: chitin/chitosan degradation, peptidoglycan/cell-wall degradation, extracellular polysaccharide synthesis, and overall carbohydrate depolymerization potential. Before index construction, the abundance of each family across all sampling sites was Z-standardized, and the standardized values were then averaged within each corresponding index.

Table S9. Composite CAZy functional indices for each soil sample.

Wildfire severity	Depth	Sample	Chitin_Deg_Index	PG_Deg_Index	EPS_Index	Carb_Depoly_Index
CK	A	CK_A_1	−1.0435	−0.0738	−1.5726	−0.8032
		CK_A_2	−0.4479	0.1788	−0.3748	−0.4094
		CK_A_3	0.1317	−0.2636	0.3197	−0.3166
		CK_A_4	−0.4497	−0.0526	−0.5412	−0.5084
	B	CK_B_1	0.7813	−0.1416	0.5612	−0.2320
		CK_B_2	0.7092	−0.1195	0.7416	0.4576
		CK_B_3	0.6483	0.0295	0.5324	0.4174
		CK_B_4	0.7116	−0.0756	0.6104	0.2107
Light	A	L_A_1	−0.6679	−0.6207	−1.1490	−0.4388
		L_A_2	−0.2265	0.2758	−0.9072	0.2192
		L_A_3	0.6393	0.0229	0.5885	0.4856
		L_A_4	−0.0826	−0.1090	−0.4906	0.0935
	B	L_B_1	0.0253	−0.1711	0.1565	−0.1502
		L_B_2	0.3412	−0.0855	0.9552	0.0801
		L_B_3	−0.1071	−0.4582	0.4312	−0.2781
		L_B_4	0.0901	−0.2374	0.5142	−0.1123
Moderate	A	M_A_1	−0.7059	0.4417	−1.0967	−0.4976
		M_A_2	−0.7083	0.4384	−0.5942	−0.2804
		M_A_3	−0.1277	0.5915	0.0337	0.1885
		M_A_4	−0.5170	0.5071	−0.5510	−0.1787
	B	M_B_1	−0.4948	0.2647	−0.6166	−0.0720
		M_B_2	−0.4526	−0.2477	−0.6190	−0.2884
		M_B_3	−0.7991	−0.4790	−0.8204	−0.3136
		M_B_4	−0.5833	−0.1559	−0.6839	−0.2029
Severe	A	S_A_1	−0.1471	−0.3612	−0.4657	−0.1021
		S_A_2	1.7369	0.6532	0.9669	1.5281
		S_A_3	−0.4754	−0.4926	−0.3418	−0.2601
		S_A_4	0.3720	−0.0676	0.0531	0.3859
	B	S_B_1	−0.0738	−0.0198	0.5231	0.1989
		S_B_2	0.8030	0.4418	1.5756	0.6503
		S_B_3	0.6604	0.1863	1.1722	0.1886
		S_B_4	0.4596	0.2004	1.0889	0.3404

Table S10. Partial-correlation matrix between composite functional indices and amino sugars/microbial necromass carbon stratified by soil layer, including FDR correction.

Depth	predictor	response	r	p	p_fdr	n	abs(r)
A	Carb_Depoly_Index	BNC	−0.5670	0.0433	0.3379	16	0.5670
A	Carb_Depoly_Index	MurA	−0.5670	0.0433	0.3379	16	0.5670
A	Chitin_Deg_Index	BNC	−0.5001	0.0818	0.3379	16	0.5001
A	Chitin_Deg_Index	MurA	−0.5001	0.0818	0.3379	16	0.5001
A	Carb_Depoly_Index	MNC	−0.4855	0.0926	0.3379	16	0.4855
A	Carb_Depoly_Index	GluN	−0.4471	0.1256	0.3379	16	0.4471
A	Carb_Depoly_Index	AG_sum_core	−0.4442	0.1283	0.3379	16	0.4442
A	PG_Deg_Index	GalN	0.4262	0.1464	0.3379	16	0.4262
A	PG_Deg_Index	FNC	0.4208	0.1522	0.3379	16	0.4208
A	Carb_Depoly_Index	FNC	−0.4203	0.1527	0.3379	16	0.4203
A	Chitin_Deg_Index	MNC	−0.4185	0.1546	0.3379	16	0.4185
A	PG_Deg_Index	AG_sum_core	0.4103	0.1638	0.3379	16	0.4103

A	PG_Deg_Index	GluN	0.4102	0.1639	0.3379	16	0.4102
A	Carb_Depoly_Index	GalN	−0.3927	0.1844	0.3379	16	0.3927
A	PG_Deg_Index	MNC	0.3917	0.1856	0.3379	16	0.3917
A	Chitin_Deg_Index	GluN	−0.3809	0.1992	0.3379	16	0.3809
A	Chitin_Deg_Index	AG_sum_core	−0.3762	0.2051	0.3379	16	0.3762
A	Chitin_Deg_Index	FNC	−0.3549	0.2341	0.3473	16	0.3549
A	PG_Deg_Index	MurA	0.3336	0.2653	0.3473	16	0.3336
A	PG_Deg_Index	BNC	0.3336	0.2654	0.3473	16	0.3336
A	EPS_Index	MurA	−0.3234	0.2810	0.3473	16	0.3234
A	EPS_Index	BNC	−0.3234	0.2811	0.3473	16	0.3234
A	Chitin_Deg_Index	GalN	−0.3208	0.2852	0.3473	16	0.3208
A	EPS_Index	MNC	−0.2362	0.4372	0.5100	16	0.2362
A	EPS_Index	GluN	−0.1985	0.5156	0.5688	16	0.1985
A	EPS_Index	AG_sum_core	−0.1927	0.5282	0.5688	16	0.1927
A	EPS_Index	FNC	−0.1732	0.5715	0.5927	16	0.1732
A	EPS_Index	GalN	−0.1352	0.6597	0.6597	16	0.1352
B	EPS_Index	BNC	−0.6946	0.0084	0.1113	16	0.6946
B	EPS_Index	MurA	−0.6945	0.0084	0.1113	16	0.6945
B	EPS_Index	MNC	−0.6717	0.0119	0.1113	16	0.6717
B	EPS_Index	AG_sum_core	−0.5898	0.0339	0.2291	16	0.5898
B	EPS_Index	GluN	−0.5724	0.0409	0.2291	16	0.5724
B	EPS_Index	GalN	−0.5376	0.0581	0.2711	16	0.5376
B	EPS_Index	FNC	−0.4924	0.0874	0.2809	16	0.4924
B	PG_Deg_Index	MNC	−0.4826	0.0949	0.2809	16	0.4826
B	PG_Deg_Index	GluN	−0.4821	0.0953	0.2809	16	0.4821
B	PG_Deg_Index	AG_sum_core	−0.4739	0.1018	0.2809	16	0.4739
B	PG_Deg_Index	FNC	−0.4638	0.1104	0.2809	16	0.4638
B	PG_Deg_Index	GalN	−0.4432	0.1293	0.3017	16	0.4432
B	PG_Deg_Index	BNC	−0.3475	0.2447	0.4625	16	0.3475
B	PG_Deg_Index	MurA	−0.3474	0.2448	0.4625	16	0.3474
B	Carb_Depoly_Index	MNC	−0.3394	0.2566	0.4625	16	0.3394
B	Carb_Depoly_Index	GluN	−0.3272	0.2751	0.4625	16	0.3272
B	Carb_Depoly_Index	AG_sum_core	−0.3236	0.2808	0.4625	16	0.3236
B	Carb_Depoly_Index	FNC	−0.3078	0.3062	0.4749	16	0.3078
B	Carb_Depoly_Index	GalN	−0.2979	0.3229	0.4749	16	0.2979
B	Chitin_Deg_Index	GalN	0.2870	0.3418	0.4749	16	0.2870
B	Carb_Depoly_Index	BNC	−0.2696	0.3730	0.4749	16	0.2696
B	Carb_Depoly_Index	MurA	−0.2696	0.3731	0.4749	16	0.2696
B	Chitin_Deg_Index	AG_sum_core	0.2462	0.4174	0.5081	16	0.2462
B	Chitin_Deg_Index	MNC	0.2309	0.4478	0.5193	16	0.2309
B	Chitin_Deg_Index	GluN	0.2231	0.4637	0.5193	16	0.2231
B	Chitin_Deg_Index	FNC	0.2102	0.4906	0.5284	16	0.2102
B	Chitin_Deg_Index	MurA	0.1825	0.5507	0.5509	16	0.1825
B	Chitin_Deg_Index	BNC	0.1824	0.5509	0.5509	16	0.1824

Note: Pearson’s partial correlations were calculated separately within each soil layer while controlling for pH, BD, and SMC. p_fdr indicates Benjamini–Hochberg FDR-adjusted P values. A = 0–20 cm; B = 20–40 cm.

Table S11. Partial-correlation matrix between enzyme activities and amino sugars/microbial necromass carbon stratified by soil layer, including FDR correction.

Depth	predictor	response	r	p	p_fdr	n	abs(r)
A	NAG	AG_sum_core	0.9978	2.31E-14	5.16E-13	16	0.9978
A	NAG	GluN	0.9977	2.95E-14	5.16E-13	16	0.9977
A	NAG	MNC	0.9957	9.89E-13	1.15E-11	16	0.9957
A	NAG	FNC	0.9954	1.52E-12	1.33E-11	16	0.9954
A	NAG	GalN	0.9921	2.87E-11	2.01E-10	16	0.9921
A	POX	MurA	0.9719	2.94E-08	1.47E-07	16	0.9719
A	POX	BNC	0.9719	2.94E-08	1.47E-07	16	0.9719
A	NAG	MurA	0.9606	1.87E-07	7.27E-07	16	0.9606
A	NAG	BNC	0.9606	1.87E-07	7.27E-07	16	0.9606

A	POX	MNC	0.9559	3.43E-07	1.20E-06	16	0.9559
A	POX	AG_sum_core	0.9382	2.11E-06	6.33E-06	16	0.9382
A	POX	GluN	0.9379	2.17E-06	6.33E-06	16	0.9379
A	CBH	GalN	0.9316	3.66E-06	9.86E-06	16	0.9316
A	POX	FNC	0.9227	7.02E-06	1.76E-05	16	0.9227
A	CBH	FNC	0.9190	9.05E-06	2.03E-05	16	0.9190
A	BG	BNC	0.9177	9.84E-06	2.03E-05	16	0.9177
A	BG	MurA	0.9177	9.84E-06	2.03E-05	16	0.9177
A	POX	GalN	0.9130	1.33E-05	2.58E-05	16	0.9130
A	CBH	AG_sum_core	0.8979	3.09E-05	5.56E-05	16	0.8979
A	CBH	GluN	0.8974	3.18E-05	5.56E-05	16	0.8974
A	CBH	MNC	0.8592	1.68E-04	2.80E-04	16	0.8592
A	BG	MNC	0.8252	5.14E-04	8.18E-04	16	0.8252
A	BG	GluN	0.7783	0.0017	0.0026	16	0.7783
A	BG	AG_sum_core	0.7777	0.0018	0.0026	16	0.7777
A	BG	FNC	0.7448	0.0035	0.0049	16	0.7448
A	CBH	MurA	0.7377	0.0040	0.0052	16	0.7377
A	CBH	BNC	0.7377	0.0040	0.0052	16	0.7377
A	BG	GalN	0.7212	0.0054	0.0068	16	0.7212
A	Cellulase	GalN	−0.1012	0.7423	0.8924	16	0.1012
A	Cellulase	FNC	−0.0738	0.8106	0.8924	16	0.0738
A	Cellulase	MurA	0.0690	0.8229	0.8924	16	0.0690
A	Cellulase	BNC	0.0689	0.8229	0.8924	16	0.0689
A	Cellulase	AG_sum_core	−0.0554	0.8575	0.8924	16	0.0554
A	Cellulase	GluN	−0.0516	0.8669	0.8924	16	0.0516
A	Cellulase	MNC	−0.0174	0.9551	0.9551	16	0.0174
B	NAG	MNC	0.9854	8.27E-10	2.89E-08	16	0.9854
B	CBH	FNC	0.9708	3.68E-08	6.44E-07	16	0.9708
B	NAG	AG_sum_core	0.9630	1.33E-07	1.55E-06	16	0.9630
B	NAG	GalN	0.9563	3.25E-07	2.84E-06	16	0.9563
B	NAG	GluN	0.9401	1.79E-06	1.25E-05	16	0.9401
B	CBH	GluN	0.9284	4.66E-06	2.72E-05	16	0.9284
B	CBH	GalN	0.9056	2.04E-05	1.02E-04	16	0.9056
B	CBH	AG_sum_core	0.8973	3.19E-05	1.40E-04	16	0.8973
B	NAG	FNC	0.8783	7.81E-05	3.04E-04	16	0.8783
B	BG	MurA	0.8411	3.14E-04	0.0010	16	0.8411
B	BG	BNC	0.8411	3.14E-04	0.0010	16	0.8411
B	NAG	MurA	0.8042	9.20E-04	0.0025	16	0.8042
B	NAG	BNC	0.8042	9.20E-04	0.0025	16	0.8042
B	CBH	MNC	0.7987	0.0011	0.0026	16	0.7987
B	BG	MNC	0.4223	0.1506	0.3513	16	0.4223
B	Cellulase	BNC	−0.3234	0.2811	0.5788	16	0.3234
B	Cellulase	MurA	−0.3234	0.2811	0.5788	16	0.3234
B	CBH	BNC	0.2954	0.3272	0.5909	16	0.2954
B	CBH	MurA	0.2954	0.3272	0.5909	16	0.2954
B	POX	FNC	−0.2893	0.3376	0.5909	16	0.2893
B	BG	AG_sum_core	0.2458	0.4183	0.6971	16	0.2458
B	POX	GluN	−0.2338	0.4420	0.7031	16	0.2338
B	BG	GalN	0.2144	0.4819	0.7224	16	0.2144
B	POX	AG_sum_core	−0.1987	0.5151	0.7224	16	0.1987
B	POX	GalN	−0.1983	0.5160	0.7224	16	0.1983
B	BG	GluN	0.1710	0.5764	0.7679	16	0.1710
B	Cellulase	FNC	0.1640	0.5924	0.7679	16	0.1640
B	POX	MurA	0.1420	0.6434	0.7767	16	0.1420
B	POX	BNC	0.1420	0.6435	0.7767	16	0.1420
B	POX	MNC	−0.1255	0.6830	0.7968	16	0.1255
B	Cellulase	GluN	0.0873	0.7768	0.8770	16	0.0873
B	Cellulase	GalN	0.0614	0.8421	0.9029	16	0.0614
B	Cellulase	MNC	−0.0478	0.8769	0.9029	16	0.0478
B	Cellulase	AG_sum_core	0.0477	0.8771	0.9029	16	0.0477
B	BG	FNC	0.0158	0.9591	0.9591	16	0.0158

Note: Pearson's partial correlations were calculated separately within each soil layer while controlling for pH, BD, and SMC. p_fdr indicates Benjamini–Hochberg FDR-adjusted P values. A = 0–20 cm; B = 20–40 cm.

Table S12. Chained indirect effects of EOC on NAG–MNC–ROC/SOC pathways.

Depth	Path	β_indirect
A	EOC → NAG → MNC	1.598
	EOC → NAG → MNC → ROC	1.269
	EOC → NAG → MNC → SOC	0.022
	EOC → NAG → MNC → ROC → SOC	1.124
B	EOC → NAG → MNC	0.543
	EOC → NAG → MNC → ROC	0.283
	EOC → NAG → MNC → SOC	-0.011
	EOC → NAG → MNC → ROC → SOC	0.243

Note: Indirect effects were calculated as the product of standardized path coefficients along each pathway. Values are provided for comparison between soil layers and were not used for causal inference. A = 0–20 cm; B = 20–40 cm.
